# Metformin improves the outcomes in Chinese invasive breast cancer patients with type 2 diabetes mellitus

**DOI:** 10.1038/s41598-021-89475-y

**Published:** 2021-05-11

**Authors:** Tianli Hui, Chao Shang, Liu Yang, Meiqi Wang, Ruoyang Li, Zhenchuan Song

**Affiliations:** Breast Center, Hebei Medical University Fourth Affiliated Hospital, No. 169 Tianshan Street, Shijiazhuang, 050035 China

**Keywords:** Cancer, Cancer, Outcomes research

## Abstract

Early reports indicate that metformin, a clinical drug administered to treat type 2 diabetes mellitus (T2DM), was found to be associated with a better prognosis of cancer. The objective of this study was retrospectively analyzed the effect of metformin on the outcomes of Chinese breast cancer patients with T2DM. A total of 3757 primary invasive breast cancer patients who underwent surgery from January 2010 to December 2013 were enrolled. According to the medication treatment, all the patients were divided as non-diabetes group, metformin group and insulin group. The follow-up data for disease-free survival (DFS) and overall survival (OS) were obtained from 3553 patients (median follow up of 85 months) and estimated with the Kaplan–Meier method followed by a log-rank test. Multivariate Cox proportional hazards regression model was applied. The results showed that there was a significant survival difference among non-diabetes group, metformin group and insulin group, 5-year DFS was 85.8%, 96.1%, 73.0%, and 5-year OS was 87.3%, 97.1%, 73.3% respectively (P < 0.05). Prognostic analysis showed metformin was significantly associated with better DFS and OS. Our results suggested that metformin may have a good effect on the survival of invasive breast cancer patients with T2DM.

## Introduction

Breast cancer is one of the most common tumors and the leading cause of cancer-related deaths in women^1^. Type 2 diabetes (T2DM) has also become a growing concern in global public health^2^. A total of 382 million people had diabetes in 2013, and this number is expected to increase to 592 million by 2035^3^. T2DM and breast cancer are quite common chronic diseases among women. Data shows that about 16% of breast cancer patients have diabetes^4^. Epidemiological studies suggest that T2DM may increase the risk of breast cancer and mortality. Women with T2DM have a 23% higher risk of breast cancer than women without T2DM and have an adverse effect on prognosis^5–7^. Recent research have found that all-cause mortality and breast cancer mortality in breast cancer patients with T2DM increased by 37% and 17%, respectively^8^.

Metformin is one of the most commonly used anti-diabetic drugs. Due to its anti-cancer properties^9^, it has become increasingly important in the treatment of breast cancer with T2DM recently. One study found that elderly breast cancer patients with diabetes had a better prognosis than those without diabetes. This might be due to the effect of metformin on breast cancer eliminates the adverse effects of diabetes on overall survival^10^. A recent study showed that the use of oral antidiabetic drug metformin can reduce the mortality of breast cancer^11^.

Although the association between T2DM and breast cancer has been extensively studied, there is a lack of research on the relationship between the treatment and control of T2DM and the prognosis of breast cancer patients, particularly in Chinese women. In this study, we retrospectively analyzed a cohort of breast cancer patients with T2DM to evaluate the relationship between the control methods of T2DM and other common clinicopathologic features and prognosis. Therefore, our study analyzed the effect of metformin on the prognosis of breast cancer patients with T2DM, and provided theoretical basis for guiding the treatment of patients and improving the overall survival rate.

## Results

### Clinicopathological characteristics

The demographic data and clinicopathological features of metformin group and insulin group were summarized in Table 1. In T2DM group, the age was older, the BMI was smaller, post-menopausal patients was more and the lymph node metastasis rate was lower than that of non-diabetes group. In addition, 112 (35.9%) patients were lymphatic metastasis-positive in the metformin group, while 43 (54.4%) in the insulin group. Besides, in the metformin group, the proportion of older patients (58.3 vs. 64.6%) and Ki-67 high expression (65.4% vs. 73.4%) were lower compared with the insulin group.

**Table 1:** Patients’ characteristics.

Characteristics n (%)	Metformin	Insulin	Non-diabetes	*P*
	n = 312	n = 79	n = 3139	
**Age at diagnosis**				0.0001
≤ 55 years	130 (41.7)	28 (35.4)	2113 (67.3)	
> 55 years	182 (58.3)	51 (64.6)	1026 (32.7)	
**BMI**				0.0001
< 25	110 (35.2)	26 (32.9)	1743 (55.5)	
25–30	150 (48.1)	39 (49.4)	1143 (36.4)	
≥ 30	52 (16.7)	14 (17.7)	253 (8.1)	
**Menopausal status**				0.0001
Pre	145 (46.5)	31 (39.2)	2168 (69.1)	
Post	167 (53.5)	48 (60.8)	971 (30.9)	
**Tumour size**				0.0179
≤ 2 cm	137 (43.9)	29 (36.7)	1499 (47.8)	
> 2 cm, ≤ 5 cm	134 (42.9)	41 (51.9)	1215 (38.7)	
> 5 cm	13 (4.2)	5 (6.3)	87 (2.8)	
Uncertain	28 (9.0)	4 (5.1)	338 (10.8)	
**Lymph node metastasis**				0.0038
0	200 (64.1)	36 (45.6)	1832 (58.4)	
1–3	79 (25.3)	25 (31.6)	744 (23.7)	
4–9	25 (8.0)	11 (13.9)	323 (10.3)	
≥ 10	8 (2.6)	7 (8.9)	240 (7.6)	
**ER status**				0.5361
Positive	244 (78.2)	59 (74.7)	2367 (75.4)	
Negative	67 (21.5)	20 (25.3)	760 (24.2)	
Unknown	1 (0.3)	0 (0)	12 (0.4)	
**PR status**				0.1507
Positive	223 (71.5)	56 (70.9)	2084 (66.4)	
Negative	88 (28.2)	23 (29.1)	1043 (33.2)	
Unknown	1 (0.3)	0 (0)	12 (0.4)	
**HER-2 status**				0.2143
Positive	58 (18.6)	16 (20.3)	692 (22.0)	
Negative	175 (56.1)	51 (64.5)	1753 (55.8)	
Uncertain	79 (25.3)	12 (15.2)	694 (22.1)	
**Ki-67**				0.0056
Low	105 (33.7)	21 (26.6)	798 (25.4)	
High	204 (65.4)	58 (73.4)	2328 (74.2)	
Uncertain	3 (0.9)	0 (0)	13 (0.4)	
**Subtype**				0.0704
ER−/PR−/HER-2−	21 (6.7)	5 (6.3)	310 (9.9)	
ER+/PR+/HER-2−	154 (49.4)	46 (58.2)	1443 (46.0)	
HER-2+	58 (18.6)	16 (20.3)	692 (22)	
HER-2 uncertain	79 (25.3)	12 (15.2)	694 (22.1)	

*BMI* body mass index, *IDC* invasive ductal carcinoma, *ILC* invasive lobular carcinoma, *ER* estrogen receptor, *PR* progesterone receptor, *HER-2* human epidermal growth factor receptor 2.

### Survival analysis

Univariate analysis showed that age, lymph node metastasis and glycemic control methods were significant factors affecting OS and DFS in breast cancer patients with T2DM (P < 0.05) (Table 2).

**Table 2:** Multivariable Cox analyses of clinical features on OS and DFS in patients with T2DM.

Evaluated factors	Multivariable (OS)		Multivariable (DFS)	
	HR (95.0% CI)	*P*	HR(95.0% CI)	*P*
**Age at diagnosis**		0.003		0.013
≤ 55 years	1		1	
> 55 years	1.302 (1.094–1.549)		1.247 (1.048–1.484)	
**BMI**		0.000		0.000
< 25	1		1	
25–30	1.280 (1.070–1.532)		1.300 (1.087–1.556)	
≥ 30	1.696 (1.300–2.213)		1.753 (1.344–2.286)	
**Tumour size**		0.000		0.000
≤ 2 cm	1		1	
> 2 cm, ≤ 5 cm	1.261 (1.053–1.511)		1.259 (1.051–1.507)	
> 5 cm	2.281 (1.602–3.247)		2.234 (1.569–3.182)	
Uncertain	1.248 (0.897–1.736)		1.241 (0.892–1.726)	
**Lymph node metastasis**		0.000		0.000
0	1		1	
1–3	2.684 (2.158–3.338)		2.716 (2.184–3.376)	
4–9	3.960 (3.072–5.105)		4.096 (3.178–5.280)	
≥ 10	9.264 (7.339–11.695)		9.837 (7.782–12.433)	
**ER status**		0.012		0.016
Negative	1		1	
Positive	0.738 (0.563–0.967)		0.738 (0.563–0.967)	
Unknown	–		–	
**PR status**		0.025		0.017
Negative	1		1	
Positive	0.745 (0.580–0.958)		0.730 (0.569–0.938)	
Unknown	–		–	
**Ki-67**		0.009		0.007
Low	1		1	
High	1.255 (1.017–1.547)		1.285 (1.042–1.584)	
Uncertain	–		–	
**Groups**		0.000		0.000
Non-diabetes	1		1	
Metformin	0.386 (0.248–0.601)		0.384 (0.247–0.598)	
Insulin	1.307 (0.848–2.014)		1.205 (0.781–1.858)	

**Figure 1:**
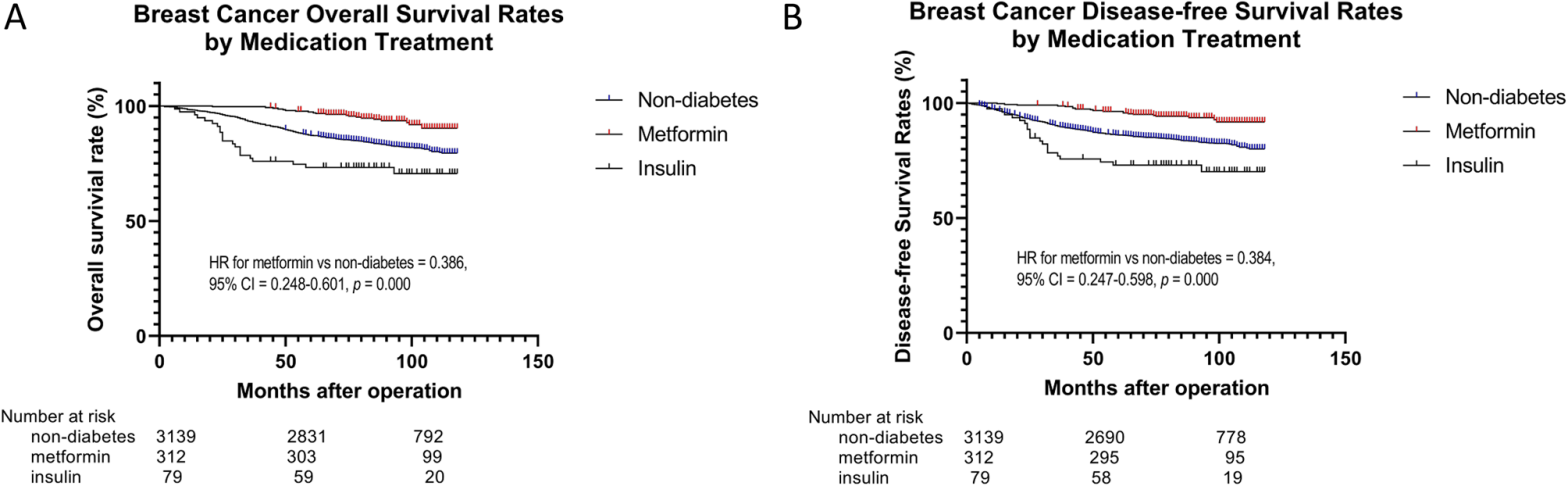
Kaplan–Meier survival estimate for OS (**A**) and DFS (**B**). *DFS* disease-free survival, *OS* overall survival.

The Kaplan–Meier OS and DFS curve using log-rank test indicated that the metformin group was significantly different from the insulin group (*P* = 0.000) (Fig. 1A,B). And metformin group had better OS and DFS than insulin group.

In multivariate analysis, metformin was significantly associated with better OS (HR 0.386, 95% CI 0.248–0.601; *P* = 0.000) and DFS (HR 0.384, 95% CI 0.247–0.598; *P* = 0.000). Meanwhile, multivariate analysis also showed that younger age, no lymph node metastasis were associated with a good prognosis (P < 0.05) (Table 2).

## Discussion

Breast cancer is the most common cancer among women worldwide and the second leading cause of cancer death^12,13^. T2DM is one of the most common chronic diseases and has become a health issue of increasing concern worldwide^14,15^. Breast cancer combined with T2DM seriously affects women's physical and mental health. Our retrospective analysis showed that metformin had beneficial effects on OS and DFS in breast cancer patients with T2DM. This is consistent with some reports in the literature. One study found that the use of metformin can reduce the all-cause mortality of breast cancer patients with T2DM^16^. A retrospective analysis pointed out that diabetic patients with breast cancer receiving metformin and neoadjuvant chemotherapy have a higher pathologic complete response rate than do diabetics not receiving metformin^17^. Another study found that patients with HER2-positive breast cancer with diabetes had better clinical outcomes after treatment with metformin than patients without metformin^18^. The study by Bayraktar et al., confirmed that although metformin did not improve the survival rate of patients with triple negative breast cancer, the trend of recurrence and metastasis was reduced compared with women without diabetes^19^. In addition, some studies have pointed out that high insulin levels are associated with increased breast cancer risk and poor prognosis. And the use of metformin can lower blood sugar and insulin levels, thereby inhibiting cancer cell proliferation and tumor growth^20,21^. In this study, it was also observed that the prognosis of the metformin group was significantly better than that of the insulin group.

How does metformin play an anti-cancer role? First, metformin can directly act on cancer cells through the protein kinase pathway activated by AMP (adenosine monophosphate), inhibiting downstream signaling of mammalian target rapamycin (a key growth factor), thereby inhibiting cell growth and proliferation^22,23^. Second, metformin can also reduce the activation of insulin/IGF-1 (insulin-like growth factor 1) receptors in tumor cells, resulting in reduced stimulation of the mitogenic pathway, thereby indirectly inhibiting cell proliferation, tumor formation and metastasis^24,25^. And recent studies have also found that metformin increased the number of CD8^+^ tumor-infiltrating lymphocytes (TILS) behavioring anti-cancer effect in the tumor microenvironment^26,27^.

As we all known, premenopausal breast cancer patients usually have a poor prognosis, but the prognosis of premenopausal patients (under 55 years old in this study) is relatively better, which may be due to the fact that most of premenopausal patients with T2DM using metformin in our study. One study pointed out that older women are less likely to receive active treatment than younger breast cancer patients, thus leading to a poorer prognosis^28^.

In conclusion, our study provided some support for the use of metformin may improve the prognosis of breast cancer patients with T2DM. However, our research has certain limitations, so these results should be carefully considered. In the future, a large number of prospective studies are needed to verify the effect of metformin on breast cancer patients to analyze the impact on the prognosis.

## Methods

### Study design and patients

A consecutive series of operable breast cancer patients from a single center was included. National guidelines were followed appropriately for the adjuvant therapy prescription. The study was approved by the Ethic Committee of the Fourth Hospital of Hebei Medical University. Informed consent for this study from all patients or relatives had been obtained during initial follow up. The present research was performed in accordance with relevant guidelines and regulations.

**Figure 2:**
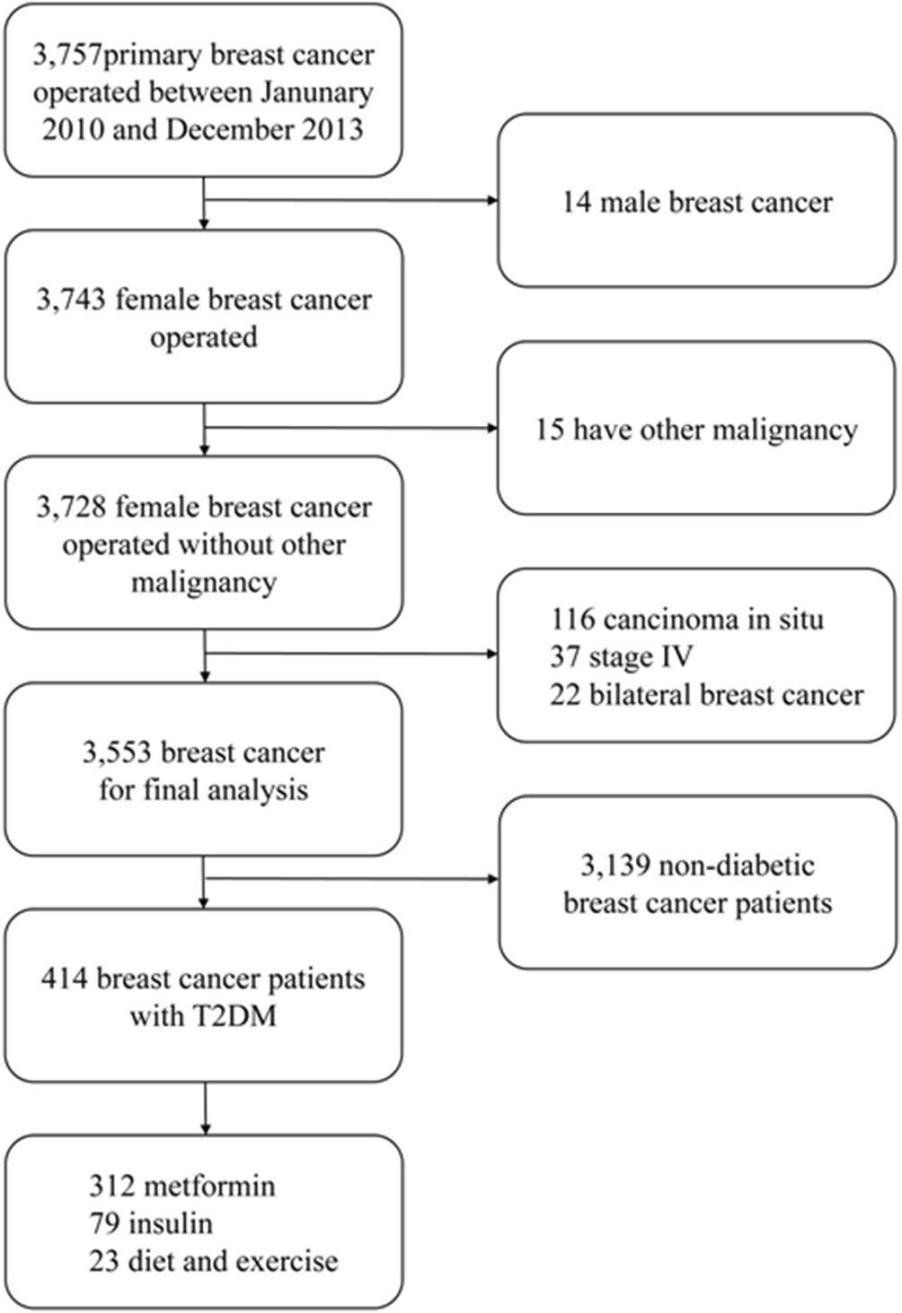
Flow chart of patient selection for final analysis.

A total of 3757 patients with primary invasive breast cancer were identified at the Breast Center of the Fourth Hospital of Hebei Medical University from January 2010 to December 2013. Patients who were diagnosed with bilateral tumors or distant metastases at the preoperative workup were excluded. A total of 3553 patients were enrolled in the final analysis, including 414 breast cancer patients with T2DM and 3139 non-diabetic breast cancer patients. We divided 414 breast cancer patients with T2DM into metformin group (312), insulin group (79), diet and exercise group (23) according to the treatment of T2DM (Fig. 2).

Oral metformin medication was defined as the metformin group, and insulin therapy was defined as the insulin group. Most patients obeyed the medication well between them during the follow-up period. However, there were a small number of patients (both no more than 10%) who were simultaneously treated with other oral hypoglycemic drugs, such as sulfonylureas (gliclazone, glimepiride), glinides (repaglinide), α glucosidase inhibitor (Acarbose), etc. Those who did not undergo drug treatment and relied on diet change and exercise enhancement were defined as diet and exercise group. And this group of patients was basically in the early stage of T2DM.

Diagnostic criteria for T2DM (according to the WHO Diabetes Expert Committee, 1999) are as follows, fasting blood glucose (FPG) ≥ 7.0 mmol/L, blood glucose 2 h after glucose load (2 h PG) ≥ 11.1 mmol/L, or random blood glucose ≥ 11.1 mmol/L^29^. The diagnosis was made based on the patient's fasting blood glucose level at the time of the first operation, history of a second-level or higher hospital diagnosis and oral hypoglycemic drugs, plus symptoms such as polyuria, polydipsia, polydipsia, weight loss.

### Tumor characteristics

Pathological information from all patients was obtained from the Department of Pathology at the Fourth Hospital of Hebei Medical University. Following American Society of Clinical Oncology/College of American Pathologists Guideline Recommendations, Estrogen receptor (ER) and progestogen receptor (PR) positive disease were defined by immunohistochemical (IHC) staining of > 1% of cells^30^. HER2-positive was defined by HER2 protein expression IHC 3+ positive and if HER2 was 2+ positive on IHC, we performed immunofluorescence hybridization (FISH) for HER2. FISH was positive if the average HER2 gene copy number ≥ 6.0 signals/cell or HER2/CEP17 ratio ≥ 2.0^31^. Ki-67 expression was categorized as low (≤ 15%) and high (> 15%). Histology type was determined according to the World Health Organization classification^32^.

### Follow-up

The starting point was the date of operation, and all ended on December 30, 2019. As we reported previously^33^. For patients who died, the date and cause of death were recorded, and all deaths not attributable to breast cancer were censored at the date of death. DFS was calculated from the date of operation to the first observed recurrence (local or distant), and patients without recurrence were censored at the time of last follow-up or death. OS was defined from the date of operation to death from any cause scored as an event. Patients who were still alive at the time of last follow-up were censored. Accordingly, the primary endpoints were DFS and OS.

### Data analysis

The SPSS 21.0 statistical software (IBM, New York City, NY) was applied for statistical analysis. The distribution of categorical variables was compared using the standard χ^2^ test between metformin and insulin groups. Survival curves were constructed with the Kaplan–Meier method, and log‐rank test for difference analysis. The univariate and multivariate Cox regression models were used to determine the association of clinical pathological features and the prognosis of different groups with DFS and OS. Hazard ratios (HRs) for DFS and OS were estimated using a Cox proportional hazards regression through multivariate analysis. Survival rates and HRs were presented with their 95% confidence intervals (CI). P < 0.05 was considered as a significant difference.
